# Psychotic-like Experiences during COVID-19 Outbreak: A survey from Pakistan

**DOI:** 10.1192/j.eurpsy.2022.654

**Published:** 2022-09-01

**Authors:** I. Ullah, F. Arain, A. Tohid, A. Ahmad, M. Jawad, A. Awan, A. Javaid

**Affiliations:** 1 Kabir Medical College Gandhara University, Psychiatry, Peshawar, Pakistan; 2 BronxCare Health System Icahn School of Medicine at Mount Sinai, Child & Adolescent Psychiatry, Bronx, United States of America; 3 University of Southern California, Psychiatry, Los Angeles, United States of America; 4 Nishtar Medical University, Psychiatry, Multan, Pakistan; 5 King Edward Medical University, Psychiatry, Lahore, Pakistan; 6 Services Institute of Medical Sciences, Psychiatry, Lahore, Pakistan; 7 Allama Iqbal Medical College, Psychiatry, Lahore, Pakistan

**Keywords:** cOVID-19, Psychosis, adolescents, psychotic-like experiences

## Abstract

**Introduction:**

Despite the fact that adolescents have been at higher risk of distress during the COVID-19 pandemic, the effect of pandemic on psychotic-like experiences (PLEs) is not well described.

**Objectives:**

The study’s objective is to evaluate if PLEs are induced in young individuals aged 18-24 during the pandemic.

**Methods:**

A total of 201 college students from Pakistan (ages 18-24) were recruited for a cross-sectional research. We investigated the incidence of PLEs in Pakistan during the pandemic, their links to socio-demographic factors, COVID-19-related characteristics, depression, anxiety, and sleep difficulties. Community Assessment of Psychic Experience’s positive symptom component (CAPE), Patient Health Questionnaire, Generalized Anxiety Disorder Scale, and IBM SPSS 25 were used.

**Results:**

CAPE-Frequency and CAPE-stress were positively associated with PHQ total (p<0.0010); GAD total (p<0.001); time spent indoors due to COVID-19 (p<0.001). Psychiatric disorder other than bipolar disorder or psychosis (p<0.001 for CAPE-frequency and stress), family history of psychiatric disorders (p<0.001 for CAPE-frequency and stress), chronic medical disease (p=0.021 CAPE-frequency and p=0.026 CAPE-stress), illegal drug usage (p<0.001 for CAPE-frequency and stress) were associated with CAPE-Frequency and CAPE-stress. In linear stepwise regression analysis, the best model predicted CAPE-Frequency explained 77.4% of variance with the following variables: PHQ total (B=0.552, SE= 0.08, t=6.909, p<0.001), GAD total (p<0.001), duration at home (p<0.001), and psychiatric disorder in family (p<0.001).

**Conclusions:**

PLEs have been linked with anxiety and depression during the pandemic. Individuals with a mental condition, family history of psychiatric disorder, chronic medical illness, illicit drug use, and increased time spent at home experienced more PLEs and stress.

**Disclosure:**

No significant relationships.

